# Host-virus interaction: the antiviral defense function of small interfering RNAs can be enhanced by host microRNA-7 *in vitro*

**DOI:** 10.1038/srep09722

**Published:** 2015-06-12

**Authors:** Xiaoying Zhang, Dongyun Liu, Sheng Zhang, Xiujuan Wei, Jie Song, Yupei Zhang, Min Jin, Zhiqiang Shen, Xinwei Wang, Zhichun Feng, Junwen Li

**Affiliations:** 1 Stem Cell Center, BaYi Children’s Hospital of The General Military Hospital of Beijing PLA, 5 Nanmencang Road, Dongcheng District, Beijing, 100700, P.R. China; 2 Departments of Neonatal Intensive Care Unit, The Affiliated Hospital of Qingdao University, 16 Jiangsu Road, Qingdao, 266003, P.R. China; 3 Department of Environment and Health, Institute of Health and Environmental medicine, Key Laboratory of Risk Assessment and Control for Environment & Food Safety, 1 Dali Road, Heping District, Tianjin, 300050, P.R. China

## Abstract

Small interfering RNAs (siRNAs) directed against poliovirus (PV) and other viruses effectively inhibit viral replication and have been developed as antiviral agents. Here, we demonstrate that a specific siRNA targeting the region between nucleotides 100–125 (siRNA-100) from the 5′-untranslated region (5′-UTR) of PV plays a critical role in inhibiting PV replication. Our data demonstrate that siRNA-100 treatment can greatly reduce PV titers, resulting in up-regulation of host microRNA-7 (miR-7), which in turn, leads to enhance inhibition of PV infection further. Moreover, our results suggest that siRNA-100 can also impair the spread of PV to uninfected cells by increasing host resistance to PV, resulting in decreasing necrosis and cytopathic effects (CPE) levels, as well as prolonging the survival of infected cells. Indeed, the active antiviral effect of siRNA-100 was potentially supplemented by the activity of miR-7, and both of them can serve as stabilizing factors for maintenance of cellular homeostasis. Results of this study identify a molecular mechanism of RNAi for antiviral defense, and extend our knowledge of the complex interplay between host and PV, which will provide a basis for the development of effective RNAi-based therapies designed to inhibit PV replication and protect host cells.

Poliovirus (PV), the etiologic agent of paralytic poliomyelitis, is a human enterovirus with a single-strand positive genomic RNA that belongs to the Picornaviridae family^1^,^2^,^3^,^4^. By 2006, indigenous transmission of PV had been interrupted in all but four countries: Afghanistan, India, Nigeria, and Pakistan^3^,^4^. However, outbreaks following PV importations into previously polio-free countries remain an ongoing risk until polio is completely eradicated^4^.

Small interfering RNAs (siRNAs), which are 19–25 nucleotides in length, mediate RNA interference (RNAi), a natural biological phenomenon regulating a wide range of cellular pathways^5^,^6^,^7^,^8^. PV has attracted intense interest as an excellent model to study RNAi^6^,^9^, and this has spurred the development of RNAi-based therapies involving anti-viral siRNAs that are capable of reducing viral yield by several orders of magnitude^6^,^9^,^10^,^11^,^12^. It remains to be determined, however, how cellular factors interact with the virus during the RNAi process and how these interactions affect PV replication and the fate of the host cells.

Besides siRNA, there is another important molecule, microRNA (miRNA), involved in the antivirus RNAi mechanism^13^. MiRNAs are evolutionarily-endogenous, regulatory noncoding RNAs that play critical roles in gene regulation^14^,^15^,^16^. Previously, we found that Hepatitis B virus (HBV) can alter host miRNA profiles, and this alteration in turn can influence the pathogenesis of HBV-relative hepatocellular carcinoma (HBV-HCC)^14^. Numerous studies have demonstrated that miRNA expression patterns are associated with virus infection, such as Epstein-Barr virus (EBV)^17^,^18^, HIV−1^19^, and human cytomegalovirus^20^, which indicates that these host miRNAs may also play important roles in host-virus interactions^18^. Furthermore, when viruses are inhibited by siRNAs, they can counter this defense by affecting host miRNA functions^21^. Thus, it is worthwhile to investigate whether an siRNA and/or miRNA pathway is involved in host-virus interactions during PV infection, and how these signal pathways could interact to affect host cellular biology.

## Results

### siRNA directed to the PV 5′-UTR reduced virus production

**Figure 1:**
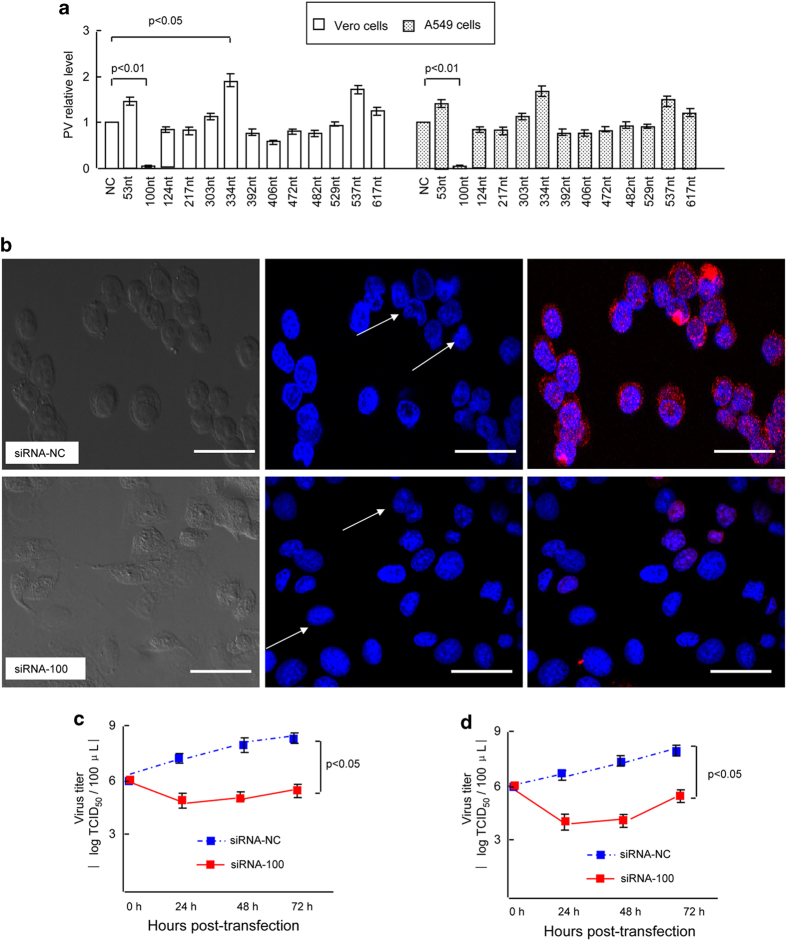
Effect of siRNA treatment targeting the 5’-UTR on PV replication. (**a**) Relative expression of PV in Vero cells transfected with 13 different siRNAs was detected using real-time PCR. All data are shown as the mean ± standard deviation based on three independent experiments and displayed as the fold change over the siRNA-NC controls. (**b**) Confocal microscopy analysis of PV in PV-infected cells. Vero cells were transfected with siRNA-NC or siRNA-100 before PV infection, as described in Experimental Procedures. Immunostaining for PV (red) was performed 24 h later using mouse anti-PV at 4 °C overnight and goat anti-mouse secondary antibody conjugated to rhodamine (TRITC). The cells were counterstained with Hoechst 33258 (nuclear, blue) and photographed using a Nikon confocal microscopy system. PV was expressed at high levels in virally infected cells transfected with siRNA-NC but at low levels in siRNA-100-treated cells. Typical nuclear CPE was observed in siRNA-NC-treated cells but not in siRNA-100-treated cells. Bars represent 20 μm. Vero cells (**c**) and A549 cells (**d**) with or without siRNA transfection were infected with PV at a TCID_50_ of 10^−6^. The cells and supernatants were harvested at different times post-transfection, and the total virus titers were determined by a TCID_50_ assay.

We designed 13 double-stranded siRNAs to target the PV 5′-UTR and assessed whether PV expression could be silenced using these specific siRNAs. The specific siRNAs were transfected into Vero or A549 cells prior to PV infection, as described in Methods, and the efficient transfection was similar (Supplementary Fig. 1). PV yield was evaluated by real-time PCR. The results revealed that an siRNA which targeted nucleotides 100–125 (siRNA-100) could significantly suppress the virus titer at least 100-fold compared with the non-targeting control siRNA (siRNA-NC), while other siRNAs produced only minor inhibitory effects (Fig. 1a). To further confirm the effect of siRNA-100, immunofluorescence and the TCID_50_ method were performed. Twenty-four hours post-infection, siRNA-100 strongly inhibited the emergence of PV in infected cells, and notably fewer viroplasms remained in the siRNA-100-treated cells, appearing as a reduced number of smaller, lighter red dots, while cells transfected with siRNA-NC were fully competent in sustaining viroplasm assembly (Fig. 1b). Moreover, the time course of PV infection showed that viral replication efficiency was significantly inhibited by transfection of cells with siRNA-100 (Fig. 1c,d).

### Effect of siRNA on the PV-induced CPE

**Figure 2:**
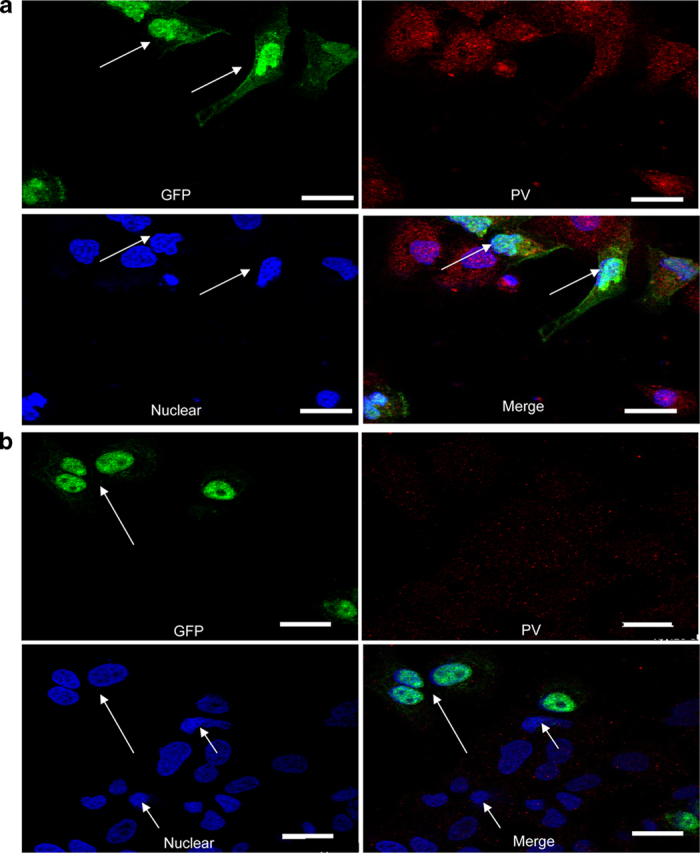
Confocal microscopy analysis of PV in PV-infected cells transfected with FAM-labeled siRNAs. Vero cells were transfected with FAM-labeled siRNA-NC (**a**) or FAM-labeled siRNA-100 (**b**). The cells exhibited a green fluorescent signal when FAM-labeled siRNAs were expressed in the cells. PV (red) was expressed at high levels in virally infected cells transfected with siRNA-NC, but at low levels in siRNA-100-treated cells. Bars represent 20 μm. PV titer: 10^−6^ TCID_50_/100 μL.

Upon infection, PV can induce a severe cytopathic effects (CPE), a typical type of death of the PV-infected cells; therefore, it was also important to determine whether siRNA-100 treatment would block the PV-induced CPE in infected cells. Cells infected with PV exhihited typical signs of CPE (i.e., nuclear deformation and partial condensation of chromatin) by 24 h post-infection with siRNA-NC transfection, as expected (Fig. 1b, arrow). However, CPE expression was greatly reduced after treatment with siRNA-100, and large proportions of the infected cells exhibited a normal nuclear appearance (Fig. 1b, arrow). Moreover, the siRNA-NC treated cells appeared as round or oval masses, and cell attachment damaged was more apparent when compared with siRNA-100-treated cells (Fig. 1b, left panel). To visualize the protective effect of the specific siRNA, we used siRNAs tagged with FAM in virally infected cells. Consistent with Fig. 1b, we found that the cells transfected with fluoresceinated siRNA-NC displayed the typical nuclear CPE (Fig. 2a, arrow), while the fluoresceinated siRNA-100-transfected cells exhibited green fluorescence and normal nuclear appearance (Fig. 2b, large arrow). Interestingly, some cells in the siRNA-100 treatment group exhibited CPE without green fluorescence (Fig. 2b, small arrow). This result further indicated that it was siRNA-100 that blocked the expression of CPE, which could protect host cells from cell death.

### Effect of siRNA on host cell biology

**Figure 3:**
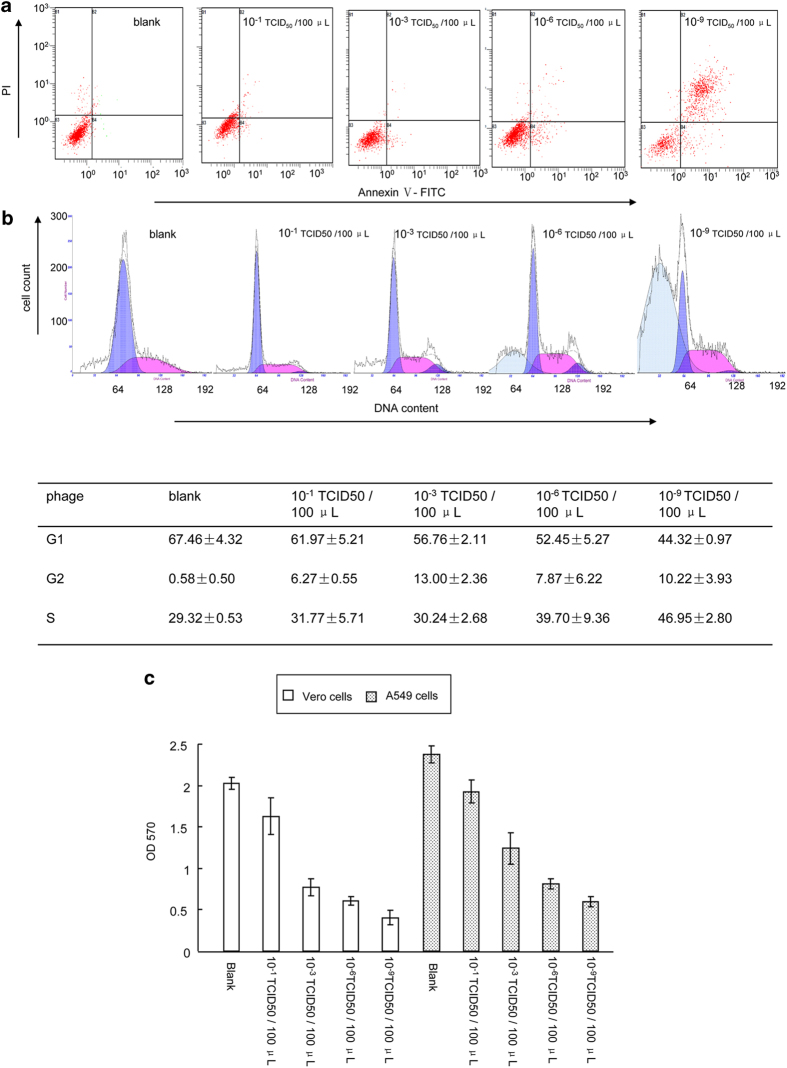
PV played a major role in cell fate decisions. (**a**) PV was involved in cell viability and necrosis, as determined using flowing cytometry. PV titers: 0, 10^−1^–10^−9^ TCID_50_/100 μL, cells: Vero cells. (**b**) PV was involved in the cell cycle, as analyzed by flow cytometry. The profiles (upper panel) were representative of at least three independent experiments. Statistical analysis was shown below. PV titers: 0, 10^−1^–10^−9^ TCID_50_/100 μL, cells: Vero cells. (**c**) PV was involved in cell growth as analyzed by MTT. PV titers: 0, 10^−1^–10^−9^ TCID_50_/100 μL, cells: Vero cells and A549 cells.

**Figure 4:**
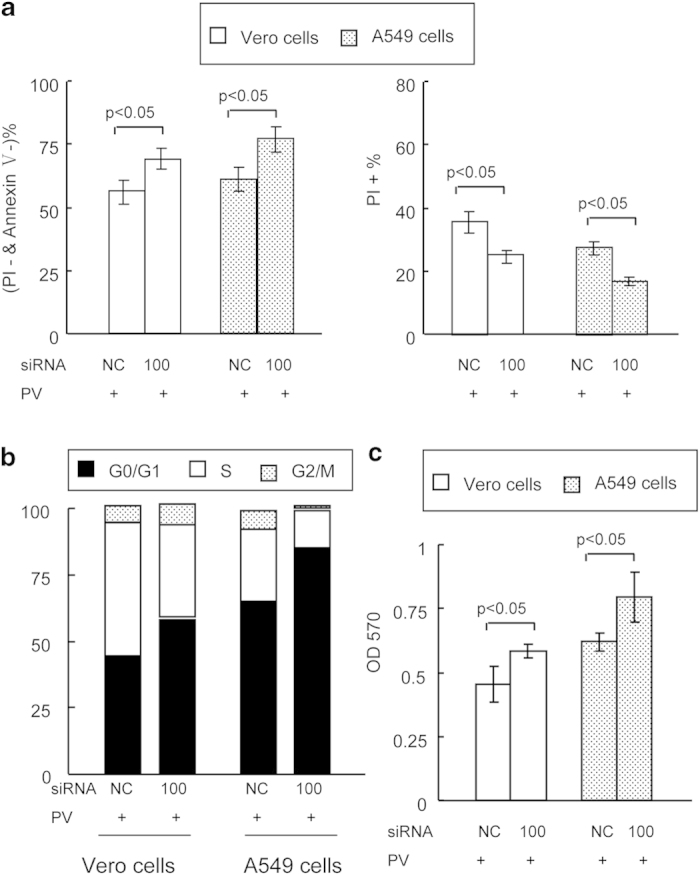
Effect of siRNA-100 on host cell biology. (**a**) siRNA-100 can significantly affect virally infected cell survival (left panel) and necrosis levels (right panel), as analyzed by flow cytometry. (**b**) siRNA-100 can significantly affect virally infected cell cycle determination, as analyzed by flow cytometry. Profiles were representative of at least three independent experiments. (**c**) siRNA-100 can significantly affect cell growth, as analyzed by MTT. PV titer: 10^−8^ TCID_50_/100 μl.

To further establish whether siRNA-100 in fact affected the biological characteristics of the host cells, uninfected and infected cells, with and without siRNA transfection, were analyzed by flow cytometry. The results demonstrated that higher PV titers (from 10^−1^ TCID_50_/100 μL to 10^−9^ TCID_50_/100 μL) induced higher levels of necrosis (from 2.49 ± 2.11% to 71.58 ± 13.77%) and reduced cell survival (from 88.90 ± 9.39% to 20.12 ± 2.66%) (Fig. 3a). Interestingly, DNA content analysis revealed that substantial fractions of cells were gradually accumulating in the S phase, and fewer were observed in the G1 phase, with increasing viral titer (Fig. 3b). Additionally, higher PV titers were correlated with reduced cell growth (Fig. 3c). This observation suggested that PV plays a major role in cell fate decisions, inducing both cell necrosis and growth arrest. Fortunately, it was evident that the siRNA-100 treatment exhibited distinct effects on cell biology compared with siRNA-NC: promoting cell survival (Fig. 4a, left panel), reducing cell necrosis (Fig. 4a, right panel), increasing G1% cells and suppressing PV-induced S-growth arrest (Fig. 4b), and enhancing cell growth (Fig. 4c). The observation that an antiviral agent can impair virus spread by prolonging the survival of infected cells^8^, implied that the specific siRNA-100 had a therapeutic effect on the host cells by increasing their resistance to virus.

### Requirement of host miR-7 for inhibiting PV replication induced by siRNA-100

**Figure 5:**
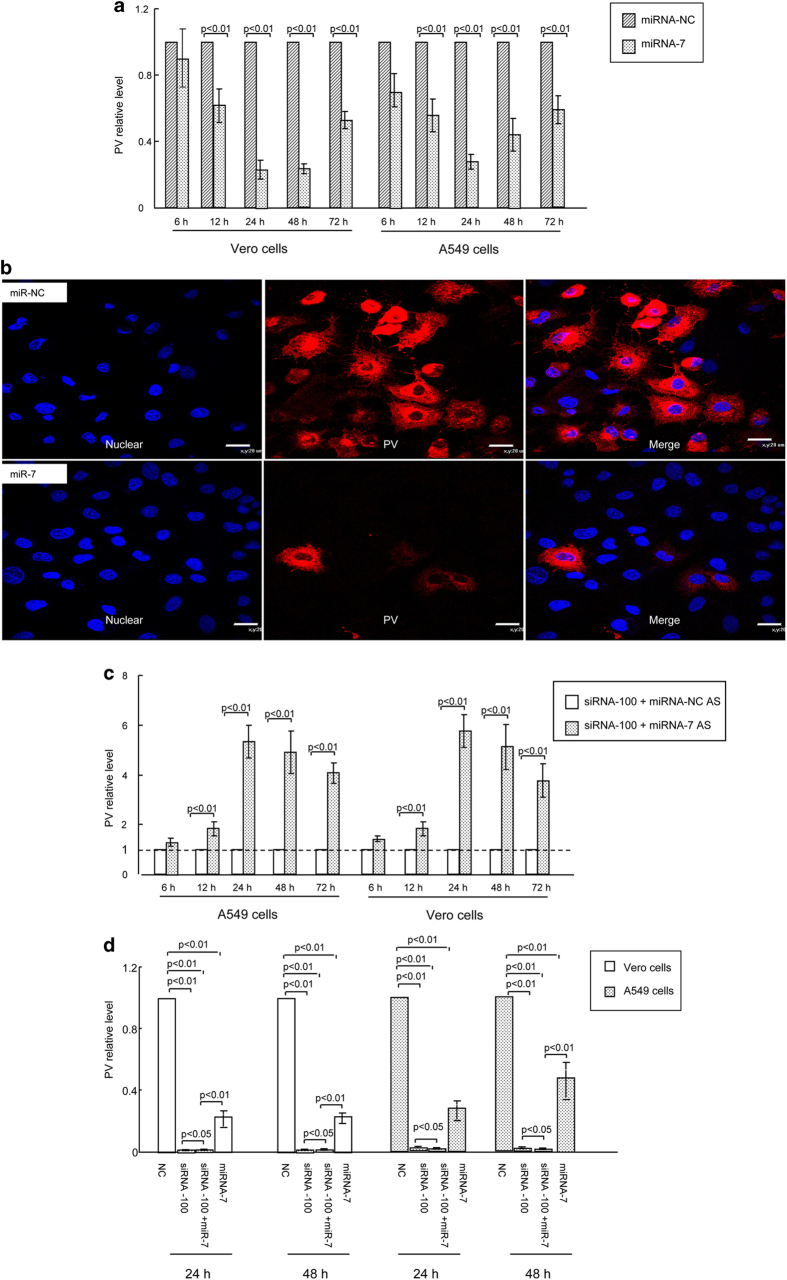
Up-regulation of host miR-7 in virally infected cells with siRNA-100 treatment. (**a**) The relative expression of PV in cells transfected with miRNA-7, or miRNA–NC control was detected by real-time PCR. (**b**) PV (red) and CPE (blue) were expressed at high levels in virally infected cells transfected with miR-NC, but at low levels in miR-7-transfected cells. Bars represent 20 μm. PV titer: 10^−8^ TCID_50_/100 μL. (**c**) The relative expression of PV in siRNA-100 and miRNA-7 AS co-transfected cells determined by real-time PCR. (**d**) The relative expression of PV in siRNA-100 and miRNA-7 co-transfected cells determined by real-time PCR. All data are shown as the mean ± standard deviation based on three independent experiments. PV titer: 10^−8^ TCID_50_/100 μL.

It has been reported that cellular miRNA can be substantially responsive to the limiting of virus replication modulated by siRNA^21^. Importantly, miRNA arrays revealed that miR-7 was significantly up-regulated in infected siRNA-100 cells compared with siRNA-NC control cells at 24 h post-infection (data not shown). Then, we used realtime PCR to confirm the microarray data and found that miR-7 was significantly up-regulated in infected siRNA-100 cells compared with siRNA-NC control cells (Table 1). To verify that the up-regulation of miR-7 was due to the decreased PV levels, we inhibited PV RNA replication using guanidine hydrochloride. Results revealed that when replication of PV was inhibited by guanidine hydrochloride, the miR-7 levels were greatly up-regulated (Table 2), which indicated that the expression levels of miR-7 were associated with PV concentration. To further investigate the interaction between miR-7 and PV, the synthesized miR-7 was transfected into PV infected cells. Results of this transfection indicated that the average transcript levels of PV was significantly lower in ectopic miR-7 expressed infected cells than in miR-NC infected cells determined by real-time PCR (Fig. 5a). As indicated in Fig. 5b, PV protein was also greatly lower in ectopic miR-7 expressed infected cells than in miR-NC infected cells, resulting in reduced CPE expression. We consistently observed that when the endogenous miR-7 was effectively blocked by miR-7 inhibitor (Antisense of miR-7, miR-7 AS, data not shown), the expression levels of PV in the siRNA-100 treated cells exhibited a significant 5-fold increase compared with cells transfected with negative control AS (NC AS) (Fig. 5c). Furthermore, Fig. 5d revealed that the levels of PV is significantly lower in siRNA-100 and miR-7 co-transfected cells than in miR-7 or siRNA-100 single transfected cells determined by real-time PCR (Fig. 5d), which indicated that siRNA-100 and miR-7 had enhanced activity together compared with their activity alone. Together, these data suggested that miR-7 contributes to the attenuation of viral replication during siRNA-100 treatment, and miR-7 can enhance inhibition of PV infection by some mechanism.

Next, to further confirm the requirement for host miR-7 in siRNA-100-induced PV inhibition, we co-transfected siRNAs that specifically deplete two core components of the miRNA machinery (Drosha and Ago2)^22^, and siRNA-100 into the cells. As expected, when Drosha and Ago2 were both silenced in siRNA-100 treated A549 cells, the miR-7 levels were significantly decreased and PV levels were significantly increased (Table 3). Taken together, these data suggested that cellular miR-7 was involved in the siRNA-100-induced suppression of PV replication.

### Effect of miR-7 on host cell biology

**Figure 6:**
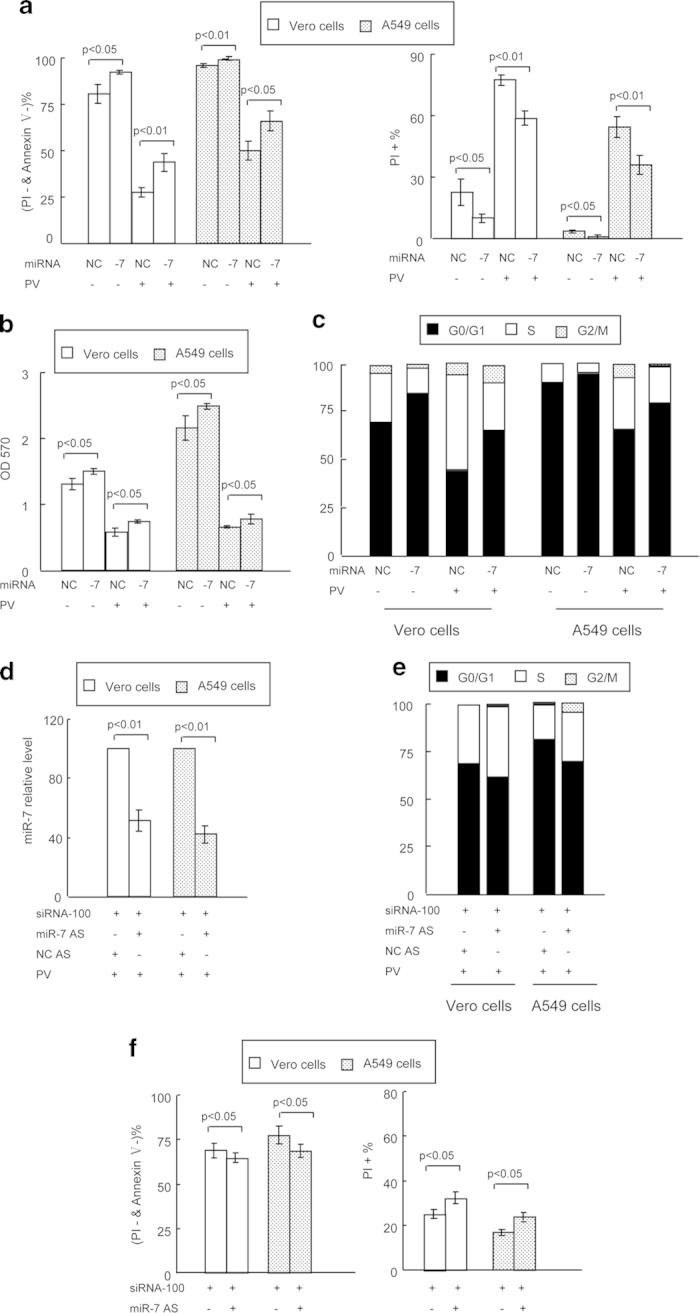
Effect of miR-7 on host cell biology. (**a**) miR-7 can significantly affect virally infected cell survival (left panel) and necrosis levels (right panel), as analyzed by flow cytometry. (**b**) miR-7 can significantly affect cell growth, as analyzed by MTT. (**c**) miR-7 can significantly affect virally infected cell cycle determination, as analyzed by flow cytometry. (**d**) Down-regulation of miR-7 in virally infected cells transfected by miR-7 AS. (**e**) The effect of miR-7 AS and siRNA-100 on cell cycle in infected cells. (**f**) The effect of miR-7 AS and siRNA-100 on cell survival (left panel) and necrosis levels (right panel) in infected cells. All data are shown as the mean ± standard deviation based on three independent experiments. PV titer: 10^−8^ TCID_50_/100 μL.

Having demonstrated that host miR-7 was required during siRNA-100-regulated PV inhibition, we wished to evaluate the effect of miR-7 on host cell biology. The synthesized miR-7 was transfected into cells with or without PV infection. Strikingly, we found that miR-7 had a small but significant effect on cell apoptosis/necrosis and viability, when the cells were not infected with PV (Fig. 6a), while it had a more pronounced effect and increased viabilities and reducing cell necrosis/necrosis levels of PV-infected cells (Fig. 6a). Additionally, the MTT assay further demonstrated that miR-7 can stimulate cell growth (Fig. 6b). As expected, the ectopic expression of miR-7 reduced the percentage of cells in the S phage, thus increasing the proportion of cells in G1, with or without PV infection (Fig. 6c).

Furthermore, to verify whether the effects of siRNA-100 on host cell viability through up-regulation of host miR-7, we co-transfected siRNA-100 and miR-7 AS into cells prior to PV infection, resulting in a corresponding 50–60% reduction in miR-7 expression levels compared with cells co-transfected with siRNA-100 and NC AS (Fig. 6d). In addition, when the host miR-7 expression levels were down-regulated, cell accumulation in the G1 phase, induced by siRNA-100, was also extenuated by miR-7 AS (Fig. 6e). Meanwhile, cell viability was reduced and cell apoptosis/necrosis increased significantly in the siRNA-100 and miR-7 AS co-transfected cells, compared with control cells co-transfected with siRNA-100 and NC AS (Fig. 6f). Collectively, these results demonstrated that siRNA-100 protected host cells from PV infection and prolonged the survival of the host cells, at least in part through up-regulation of host miR-7 expression. These results suggested that miR-7 plays an important role in host-virus interaction, acting as a rheostat to maintain cellular homeostasis.

## Discussion

**Figure 7:**
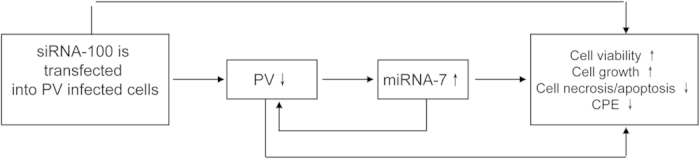


Recently research has indicated that RNAi, mediated by siRNAs or miRNAs, is an evolutionarily conserved mechanism that plays an important role in inhibiting the replication of viruses. However, most studies on the roles of RNAi in host-virus interactions have been carried out separately, using either siRNAs or miRNAs. Information regarding the expression profiles of host miRNAs altered by siRNAs in virus infection is very limited. In the current study, we found that host miR-7 can be induced by down-regulation of PV inhibited by siRNA-100, and the effective antiviral effect was potentially supplemented by the activity of miR-7, which may function by altering the cellular environment to be further inhibitory for viral replication, resulting in creating an interesting positive feedback response (Fig. 7).

siRNA-mediated silencing of viral genes has been employed to inhibit the replication of a variety of DNA and RNA viruses, targeting both noncoding and coding sequences, *in vitro* and *in vivo*^8^,^10^,^11^,^12^,^23^,^24^. However, viral escape subsequent to exposure to antiviral siRNAs has also been observed in several studies^25^,^26^,^27^. Thus, selecting specific siRNAs targeting highly conserved regions of the viral genome, such as the 5′-UTR, can result in effective inhibition of viral replication^28^. However, siRNAs that targeted the 5′-UTR of picornavirus failed to reduce virus replication^29^, whereas Pelletier *et al.* found that a combination of two synthetic siRNAs targeting both the 5′-UTR and the 3D polymerase of PV was more effective and persistent than a single siRNA^6^. Additionally, in our previous study, we found that the PV 5′-UTR was a critical region for its disinfection by chlorine dioxide^30^. Interestingly, in the present study, we found that a specific siRNA, targeting the region between nucleotides 100–125 of the PV 5′-UTR strongly inhibited viroplasm formation in the context of PV infection and blocked PV-induced cell killing in virally infected Vero and A549 cells. It is known that the 5′-UTR of the PV genome is mainly associated with viral transcription, replication, translation, and invasion. The 5′-UTR has six stem-loop domains, representing two functional elements: (1) Domain I is the 1–80 nt region that forms a cloverleaf structure and is associated with the replication of viral nucleic acids. (2) Domains II–VI, in the 130–610 nt region, are associated with the synthesis of viral proteins^6^,^25^,^30^,^31^. In view of the fact that the specific siRNA-100 target sequence is located between nucleotides 100–125, which itself falls between Domains I and II, we hypothesized that this may help to prevent the stem-loop secondary structures from shielding the viral target RNAs access to the RNAi machinery.

Our results indicated that cellular miR-7 can be induced by down-regulation of PV inhibited by siRNA-100, and may be implicated in viral infection of mammalian cells with antiviral potential. It has been reported that miR-7 is widely conserved in animal species and may be induced in response to infections of mammalian viruses, including adenovirus, influenza A virus and HBV^18^,^32^,^33^,^34^. Chen *et al.* reported that the HBV × protein (HBX) up-regulated miR-7 expression to target the epidermal growth factor receptor (EGFR), which in turn modulated HBV-HCC cellular behavior^34^. It has also been reported that miR-7 can act as an antiviral factor by interacting with the genes of white spot syndrome virus (WSSV), wsv477, an early gene involved in viral DNA replication^18^. Importantly, in our study, we discovered that siRNA-100 can inhibit PV replication, resulting in up-regulation of host miR-7, and the up-regulation of cellular miR-7 can significantly decrease the yield of PV further, creating an effective positive feedback response (Fig. 7). While careful interpretation of this observation is required, these results suggest that the “siRNA-100–PV–miR-7” interaction plays an important antiviral defense role during PV infection.

Furthermore, increased G1% cells and decreased S% cells were observed more extentively in siRNA-100-treated cells when compared with siRNA-NC-treated cells (Fig. 4b), and G1% cells increase was also observed following induction of miR-7 activity in cells (Fig. 6c), which was consistent with Sanchez *et al.*^15^. And G1 arrested cells were reported to be associated with reduced virus infection^35^, and we also observed the phenomenon (Supplementary Fig. 2), which indicated that the activity of inhibitors of PV replication, either chemical or siRNA-100, was highly antiviral and this effect was potentially supplemented by the activity of miR-7, which may function by altering the cellular environment to be further inhibitory for viral replication. Additionally, the decreased apoptosis/necrosis levels and increased cell viability in siRNA-100-treated cells that we observed were likely due to lower PV titers and higher miR-7 activity in siRNA-100-treated cells than in siRNA-NC-treated cells. However, the effects of miR-7 on cell growth and apoptosis have been the subject of some controversy. Sanchez and colleagues previously reported that miR-7 had no effect on cell apoptosis of CHO cells^15^. However, Xiong and colleagues found that miR-7 can significantly inhibit cell growth and induce cell apoptosis via targeting of BCL-2^36^. Additionally, Saydam and colleagues reported that over-expression of miR-7 can reduce cell growth through modulation of EGFR and p21-activated kinase 1 (Pak1)^37^. Interestingly, Chou *et al.* reported that EGFR stimulated miR-7 expression, and the over-expression of miR-7 promoted cell growth via targeting of ERF^38^. Moreover, Cheng *et al.* reported that inhibition of miR-7 down-regulated cell growth and up-regulated apoptosis levels^39^. Therefore, these data indicated that the role of miR-7 may be different in different environments. In this study, we observed that up-regulated miR-7 can significantly inhibit cell apoptosis and induce cell growth, which contributed to the antiviral defense of the host cells. Both apoptosis and necrotic death are known to serve as an important defense mechanisms to eliminate viruses, but when necrosis or apoptosis are excessive, they can potentially cause irreversible cellular lysis, tissue damage, and death of the whole organism^40^,^41^. Our results indicated that siRNA-100 treatment can significantly promote cellular resistant to PV partly through regulation of host miR-7, resulting in prolonged survival, reduced apoptosis/necrosis levels and reduced levels of CPE in infected cells (Figs. 4, 5, 6). Thus, the antiviral defense is not acting through the induction of apoptosis and necrotic death in infected cells. Based on the results of this study, we believe that impairing viral spread to uninfected cells by prolonging the survival of infected cells represents a potential effective therapeutic strategy. These findings will also extend our knowledge of the complex interplay between a virus and its host. Both siRNA-100 and miR-7 appear to act as stabilizing factors for maintaining of cellular homeostasis, which is consistent with previous reports that miR-7 may enter into novel genetic relationships to buffer developmental programs against variation, and impart robustness during environmental perturbations^15^,^16^,^42^. Although the details regarding how siRNA-100, miR-7 and other host factors cooperate in antiviral responses need to be further clarified, it will be a clinically interesting issue to address how cells are protected, and how they avoid acute or chronic inflammation associated with necrosis induced by PV.

## Methods

### Cells and virus

African green monkey kidney cells (Vero cells) and human lung adenocarcinoma epithelial cells (A549 cells) were used as host cells and cultured in RPMI-1640 with 10% fetal bovine serum. Poliovirus type 1 strain LSc 2ab used in the study was titrated by the 50% tissue culture infectious dose (TCID_50_) method as previously described^30^.

### siRNAs

The different choices of the 25-nt-long sequences were made with the assistance of the GenePharma program ( http://www.genepharma.com) according to the corresponding 5’-UTR segments of the genome of PV type 1 strain LSc 2ab from 1–600 nt (Table S1). Oligoribonucleotides were synthesized by GenePharma and used at a concentration of 20 μM. The siRNA sequences used to target the human Ago2 and Drosha (Table S1)^22^ were synthesized by GenePharma and used at a concentration of 20 μM.

### miRNAs

The miRNA mimics and miRNA inhibitors were synthesized by GenePharma and used at a concentration of 20 μM. Sequences were listed in Table S1

### Transfection and infection of cells

Transfections were performed using a Lipofectamine^™^ 2000 kit (Invitrogen,Carlsbad, CA) according to the manufacturer’s instructions and our previous report^14^. Cells were transfected with double-stranded siRNA or miRNA mimics or negative mock controls. At 24 h post-transfection, the cells were infected with PV for 30 min at different initial virus concentrations. The infected cells were washed with serum-free medium and then harvested for the subsequent experiments after incubating in fresh medium at 37 °C for 24 h.

### Reverse transcription and real-time PCR analysis

Total RNAs were purified with the mirVana^™^ PARIS^™^ Kit (Ambion, CA, USA). Reverse transcription (RT) reactions and real-time PCR for *PV* has been described^14^,^18^. Relative expression of *PV* was calculated with the 2^−ΔΔCt^ method^14^,^18^. The primers were listed in Table S1. RT reactions and real-time PCR for *mir-7* were performed using TaqMan Small RAN Assay (ABI, CA, USA)^43^, and TaqMan CT values were converted into absolute copy numbers using a standard curve from synthetic mir-7 mimics^43^.

### Measurement of cell apoptosis by flow cytometry

Cells transiently transfected with siRNA or miRNA were plated in 6-well plates with or without siRNA-100 transfected, then the PV at different titers was added at 24h post-trasfection. After post-infection for 24 h, the cells’ apoptosis was measured by ApopNexin^™^ FITC Apoptosis Detection Kit (APT750, Millipore, Temecula, CA) as we previously described^44^. Briefly, cells were harvested and spun down (400 g, 5 min) and washed by PBS, resuspending cells in 1× bind buffer at a concentration of 10^6^ cells/ml. Then, took 200 μl cell suspension and added 3 μl of the annexin conjugate ApopNexin™ FITC and 2 μl PI. Finally, samples were mixed and incubated for 15 min at room temperature in the dark and placed the cells on ice. Fluorescence due to FITC and PI staining was measured in a flow cytometer (Cytomics FC 500, Beckman Coulter).

### Measurement of cell cycle by flow cytometry

Uninfected and infected cells with or without siRNA/miRNA transfection were harvested by trypsinization. After centrifugation, cells were fixed in 70% ethanol at −20 °C overnight and then resuspended in binding buffer containing 20 mg/ml propidium iodide and 0.5 mg/ml RNase at 37 °C for 30 min before analysis by a flow cytometry (Cytomics FC 500, Beckman Coulter). Cell-cycle compartments were integrated by a Multicycle Program.

### Cell proliferation assays

Uninfected and infected cells with or without siRNA/miRNA transfection were seeded in 96-well plates. Assessment of cell proliferation was measured in terms of optical absorbance (OD) per well by a semiautomated tetrazolium-based colorimetric assay using 3-(4,5-dimethylthiazol-2-yl)-2,5-diphenyltetrazolium bromide (MTT, Amresco) according to the manufacturer’s instructions^45^. Optical density was read with a microplate reader (Bio-Rad) at a 570 nm wavelength.

### Immunofluorescence and confocal microscopy

Immunostaining for PV (antibody used at 1:50 dilution; Abcam) and nuclear DNA (Hoechest 33258, 0.1%; Sigma, St. Louis, USA) were performed as previously described^14^, and imaged using a confocal laser scanning microscope (Nikon, C1 si; Japan).

### Statistical analysis

Data are presented as means ± SD. All data were analyzed with SPSS for Windows version 17.0 (SPSS Inc., Chicago, IL, USA). One-Way ANOVA analyses and the LSD test were used to compare different groups. Differences were deemed statistically significant if they had p values less than 0.05.

## Additional Information

**How to cite this article**: Zhang, X. *et al*. Host-virus interaction: the antiviral defense function of small interfering RNAs can be enhanced by host microRNA-7 *in vitro*. *Sci. Rep.* **5**, 9722; doi: 10.1038/srep09722 (2015).

## Supplementary Material

Supplementary Information

## Figures and Tables

**Table 1:** The expression of miR-7 in infected cell transfected with siRNA-100 or siRNA-NC was determined by realtime PCR (molecules per cell).

post-transfection hours:		24 h	48 h	72 h
Vero cells	siRNA-NC	11.65 ± 0.04	5.74 ± 0.30	1.71 ± 0.42
	siRNA-100	74.44 ± 6.03**	40.25 ± 1.69**	4.61 ± 1.22*
A549 cells	siRNA-NC	5.48 ± 0.10	3.90 ± 0.35	0.29 ± 0.02
	siRNA-100	45.80 ± 2.41**	17.90 ± 0.44**	1.51 ± 0.06**

^**^p < 0.01, compared with siRNA-NC;

^*^p < 0.05, compared with siRNA-NC.

**Table 2:** The expression of PV and miR-7 in infected cells treated with guanidine HCl was determined by realtime PCR.

	Vero cells			A549 cells		
guanidine HCl (mM)	0	0.2	2.0	0	0.2	2.0
PV (∆CT values)	−7.12 ± 0.84	−3.54 ± 0.31**	NT**,§§	−6.32 ± 0.40	−4.91 ± 0.38**	NT**,§§
miR-7 levels (molecules per cell)	10.84 ± 0.34	35.55 ± 5.39**	187 ± 11.84**,§§	5.55 ± 0.19	12.52 ± 0.56*	59.89 ± 5.43**,§§

NT: PV was not tested;

**p < 0.01 compared with 0 mM guanidine HCl;

*p < 0.05 compared with 0 mM guanidine HCl;

^§§^p < 0.01 compared with 0.2 mM guanidine HCl;

**Table 3:** The expression of PV and miR-7 in infected A549 cell co-transfected with siRNA-100 plus siRNA-NC, or co-transfected with siRNA-100 plus siRNA-ago and siRNA-drosha, was determined by realtime PCR.

post-transfection hours:		24 h	48 h	72 h
PV (∆CT values)	siRNA-100 + siRNA-NC	0.23 ± 0.06	0.10 ± 0.06	−2.16 ± 0.12
	siRNA-100 + siRNA-ago + siRNA-drosha	−3.19 ± 0.14**	−3.07 ± 0.18**	−3.89 ± 0.21*
miR-7 levels (molecules per cell)	siRNA-100 + siRNA-NC	44.07 ± 4.86	17.80 ± 0.84	0.32 ± 0.07
	siRNA-100 + siRNA-ago + siRNA-drosha	5.69 ± 0.27**	5.01 ± 0.25**	0.13 ± 0.03**

**p < 0.01, compared with siRNA-100 + siRNA-NC;

*p < 0.05,compared with siRNA-100 + siRNA-NC.
