# Supplementary material for: Host-virus interaction: the antiviral defense function of small interfering RNAs can be enhanced by host microRNA-7 in vitro
Source: Sci Rep. 2015 Jun 12;5:9722. doi: 10.1038/srep09722 (PMC4464290; doi:10.1038/srep09722)
Supplement: Supplementary Information [file srep09722-s1.pdf]

Host-virus interaction: the antiviral defense function of small interfering RNAs can be enhanced by host microRNA-7 in vitro

Xiaoying Zhang[1,*], Dongyun Liu[2,*], Sheng Zhang[1], Xiujuan Wei[1], Jie Song[1], Yupei Zhang[1], Min Jin[3], Zhiqiang Shen[3], Xinwei Wang[3], Zhichun Feng [1, †], Junwen Li [3, †]

[*] These authors contributed equally to the manuscript.

[1]Stem Cell Center, BaYi Children's Hospital of The General Military Hospital of Beijing PLA, 5 Nanmencang Road, Dongcheng District, Beijing, 100700, P.R.China.

[2]Departments of Neonatal Intensive Care Unit, The Affiliated Hospital of Qingdao University. 16 Jiangsu Road，Qingdao, 266003, P.R. China.

[3]Department of Environment and Health, Institute of Health and Environmental medicine, Key Laboratory of Risk Assessment and Control for Environment & Food Safety, 1 Dali Road, Heping District, Tianjin, 300050, P.R. China.

[†]**Corresponding author**: Prof. Zhichun Feng and. Prof. Junwen Li. Prof. Zhichun Feng: 5 Nanmencang Road, Dongcheng District, Beijing, 100700, P.R.China. Phone: (86)10-66721786; Fax: (86)10-64063099; E-mail: fengzhichun81@sina.com. Prof. Junwen Li: 1 Dali Road, Heping District, Tianjin, 300050, P.R. China. Phone: (86)22-84655345; Fax: (86) 22-84655345; E-mail: junwen9999@hotmail.com

Supplementary Table 1. Oligonucleotide Sequences for siRNA and target genes.

	Name	Sequence
siRNA		
5'UTR-53nt	Sense	5’-acuccgguauugcgguacccuuguatt-3'
	Antisense	5’-uacaaggguaccgcaauaccggagutt-3'
5'UTR-100nt	Sense	5’-guaacuuagacgcacaaaaccaagutt-3'
	Antisense	5’-acuugguuuugugcgucuaaguuactt-3'
5'UTR-124nt	Sense	5’-guucaauagaagggggguacaaaccatt-3'
	Antisense	5’-ugguuuguaccccccuucuauugaactt-3'
5'UTR-127nt	Sense	5’-gacggauccguuauccgcuuauguatt-3'
	Antisense	5’-uacauaagcggauaacggauccguctt-3'
5'UTR-303nt	Sense	5’-gaguguagcuuaggcugaugagucutt-3'
	Antisense	5’-agacucaucagccuaagcuacacuctt-3'
5'UTR-334nt	Sense	5’-cccucaccggugacgguggucc aggtt-3'
	Antisense	5’-ccuggaccaccgucaccggugagggtt-3'
5'UTR-406nt	Sense	5’-gaacaaggugugaagagccuauugatt-3'
	Antisense	5’-ucaauaggcucuucacaccuuguuctt-3'
5'UTR-472nt	Sense	5’-aaccucggagcagguggucacaaactt-3'
	Antisense	5’-guuugugaccaccugcuccgagguutt-3'
5'UTR-482nt	Sense	5’-cagguggucacaaaccagugauuggtt-3'
	Antisense	5’-ccaaucacugguuugugaccaccugtt-3'
5'UTR-529nt	Sense	5’-uggcggaaccgacuacuuugggugutt-3'
	Antisense	5’-acacccaaaguagucgguuccgccatt-3'
5'UTR-537nt	Sense	5’-ccgacuacuuuggguguccguguuutt-3'
	Antisense	5’-aaacacggacacccaaaguagucggtt-3'
5'UTR-617nt	Sense	5’-cgaauuggauuggccauccggugaatt-3'
	Antisense	5’-uucaccggauggccaauccaauucgtt-3'
Ago2	Sense	5’- ggaaaaugaugcugaauauuu-3’
	Antisense	5’-auauucagcaucauuuuccuu-3’
Drosha	Sense	5’-gaguaggcuucgugacuuauu-3’
	Antisense	5’-uaagucacgaagccuacucuu-3’
Negative	Sense	5’-uucuccgaacgugucacgutt-3’
Control	Antisense	5’-acgugacacguucggagaatt-3’
miRNA		
miR-7	Sense	5’-uggaagacuagugauuuuguugu-3’
	Antisense	5’-aacaaaaucacuagucuuccauu-3’
miR-NC	Sense	5’-uucuccgaacgugucacgutt-3’
	Antisense	5’-acgugacacguucggagaatt-3’
miR-7 AS		5’-acaacaaaaucacuagucuucca-3’
NC AS		5’-uuguacuacacaaaaguacug-3’
Primers		
miR-7	Stem-loop	5’-gtcgtatccagtgcgtgtcgtggagtcggcaattgcactgga tacgacacaacaa-3’

	Sense	5’-cggtggaagactagtgatt-3’
	Antisense	5’-tgcgtgtcgtggagtc-3’
u6	Sense	5’-gcttcggcagcacatatactaaaat-3’
	Antisense	5’-cgcttcacgaatttgcgtgtcat-3’
PV	Sense	5’- ggctgcttatggtgacaatcacag-3’
	Antisense	5’- gtggtgtaattaatggtagaaccacc-3’
GAPDH (VERO)	Sense	5’-cgggaaactgtggcgtgat-3’
	Antisense	5’-gggtgtcgctgttgaagtcg-3’
GAPDH (A549)	Sense	5’-gaccactttgtcaagctcatttcc-3’
	Antisense	5’-gtgagggtctctctcttcctcttgt-3’

Supplementary Fig 1

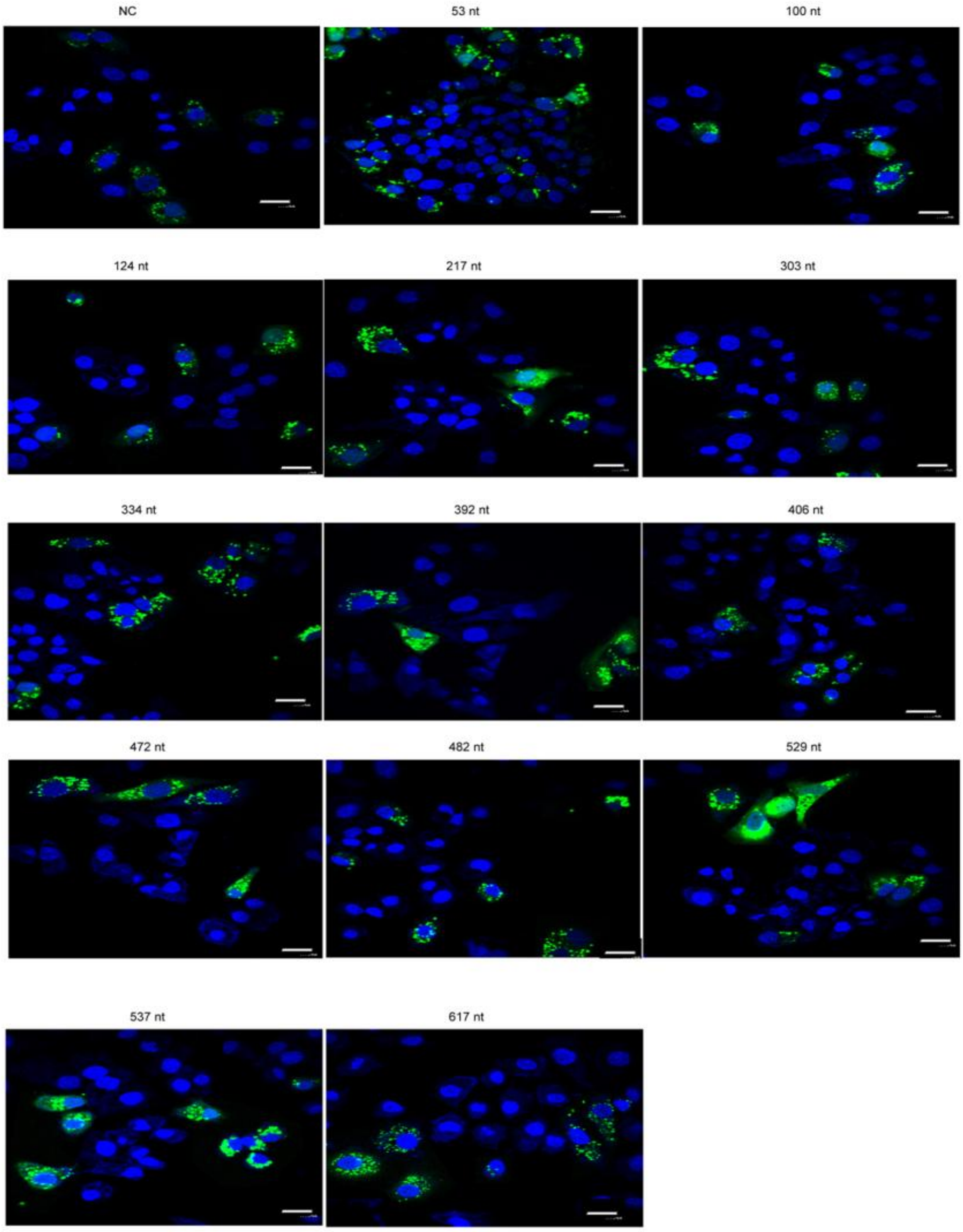

Supplementary Fig 2

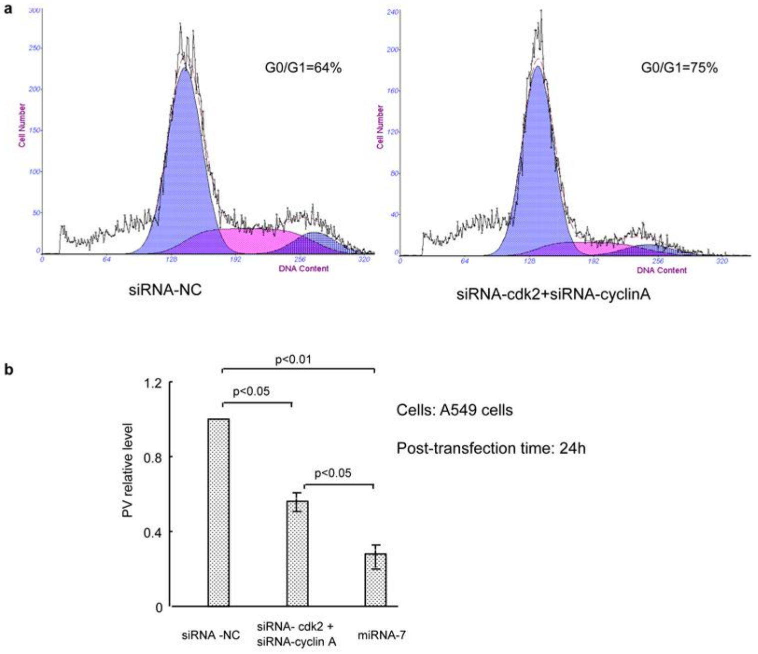

Supplementary Figure legends

Supplementary Fig 1 Confocal microscopy analysis of efficient transfection of all the FAM-labeled siRNAs. A549 cells were transfected with FAM-labeled siRNA-NC or FAM-labeled siRNAs, and displayed similar green fluorescent signal. Bars represent 20 μm.

Supplementary Fig 2 G1 arrested cells showed reduced PV infection. (a) A549 cells are arrested in G1 by siRNA directed against cdk2 (gene ID: 1017, target sequence: AGGTGGTGGCGCTTAAGAAAA) and cyclin A (gene ID: 890, target sequence: GCCAGCTGTCAGGATAATAAA) before PV infection as analyzed by flow cytometry. (b) The relative expression of PV in

different siRNA transfected cells determined by real-time PCR. PV titer: 10^{-8} $TCID_{50}$/100 μL.
